# In vitro investigation of chemical properties and biocompatibility of neurovascular braided implants

**DOI:** 10.1007/s10856-019-6270-6

**Published:** 2019-06-04

**Authors:** Giorgio Cattaneo, Chris Bräuner, Gerd Siekmeyer, Andreas Ding, Sabina Bauer, Markus Wohlschlögel, Lisa Lang, Teresa Hierlemann, Maria Akimov, Christian Schlensak, Andreas Schüßler, Hans-Peter Wendel, Stefanie Krajewski

**Affiliations:** 1 Acandis GmbH, Pforzheim, Germany; 2 Admedes GmbH, Pforzheim, Germany; 3 Acquandas GmbH, Kiel, Germany; 4 Department of Thoracic and Cardiovascular Surgery, Clinical Research Laboratory, University Medical Center, Tuebingen, Germany

## Abstract

Braiding of Nitinol micro wires is an established technology for the manufacturing of fine-meshed neurovascular implants for tortuous vessel geometries. Electropolishing of wires before the braiding process has the potential to improve the in vitro behaviour in terms of thrombogenicity and endothelial cell proliferation. In this study, we present the first in vitro investigation of braided electropolished/blue oxide Nitinol samples in a blood flow loop, showing a significantly lower activation of the coagulation pathway (represented by the TAT III marker) and a tendency towards reduced platelet adhesion. Furthermore, we applied the same surface treatment on flat disks and measured protein adhesion as well as endothelial cell proliferation. We compared our results to non-electropolished samples with a native oxide surface. While platelet deposition was reduced on electropolished/blue oxide surface, a significant increase of endothelial cell seeding was observed. Investigation of inflammatory marker expression in endothelial cells provided divergent results depending on the marker tested, demanding closer investigation. Surface analysis using Auger electron spectroscopy revealed a thin layer mainly consisting of titanium oxynitride or titanium oxide + titanium nitride as a potential cause of the improved biological performance. Translated to the clinical field of intracranial aneurysm treatment, the improved biocompatibility has the potential to increase both safety (low thrombogenicity) and effectiveness (aneurysm neck reconstruction).

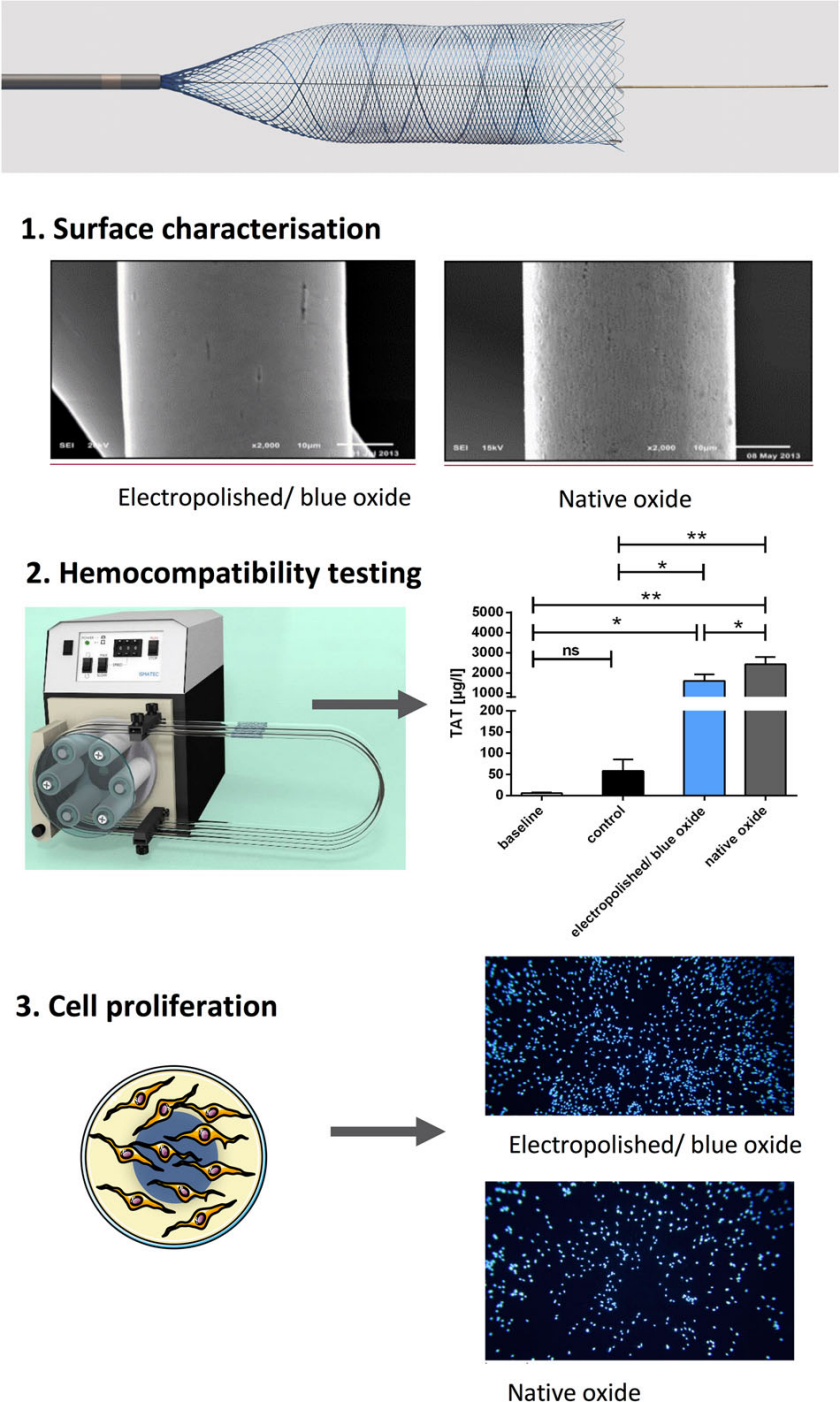

## Introduction

In the last three decades, braided stents have become established devices for endovascular treatment of blood vessel disease. Starting in 1986 with the introduction of the Wallstent™ endoprosthesis for treatment of coronary artery stenosis [1], braided stents nowadays find broad application in the treatment of carotid artery [2] and peripheral vessel diseases such as in superficial femoral and popliteal arteries [3, 4]. The implant structure consisting of several interwoven wires has unique features compared to laser-cut stents. A considerable implant deformation, resulting in an enhanced flexibility in curved vessel anatomies and radial compression in delivery catheters, is achieved by a relative sliding of the wires at crossing points. Furthermore, the use of small wires with tight manufacturing tolerances allows for the realization of fine-meshed, low porosity structures designed for blood filter or blood flow modulation.

In the last 10 years, these unique features have led to a growing application of the braided technology in the neurovascular field, where implant delivery through miniaturized microcatheters with inner diameter between 0.0165″ and 0.021″ and deployment in small vessels with tortuous anatomy are required. Beside laser-cut stents, braided stents are widely used for coil stabilization in intracranial aneurysm embolization procedures (stent-assisted coiling) [5, 6]. Moreover, enhanced mesh density led to the introduction of the novel therapeutic concept of “flow diverter” treatment, with the aim of achieving long-term intraaneurysmal occlusion by means of blood flow redirection and vessel remodelling [7].

Among biocompatible materials, the nickel titanium alloy Nitinol represents a gold standard for neurovascular implants, because of its superelastic behaviour in a high deformation range. However, while the Nitinol surface of laser-cut stents is generally electropolished after the cutting process and thermal treatment, braided implants are normally not electropolished as a final manufacturing step. In fact, electropolishing of braided implants is complicated due to wire overlapping, resulting in inhomogeneity of the material removal and loss of stability at wire crossing points. Otherwise, not-polished surfaces are thought to display a higher impurity [8] and a thicker, porous oxide layer, which is usually less protective in terms of nickel ion release and corrosion. However, wire electropolishing as a manufacturing step before the braiding process has the potential to provide a homogeneous surface with properties similar to laser-cut stents.

In this study, we compared the thrombogenicity of braided implants with pre-electropolished wires with design-like, but not electropolished, implants in an in vitro blood flow model. Furthermore, we investigated blood protein adsorption as well as endothelial cell adhesion and expression of various inflammatory markers on electropolished and non-electropolished disks.

## Materials and methods

### Test specimens

Two different test specimens were used for this study. For the surface characterization and in vitro thrombogenicity model, braided implants were manufactured, corresponding to the design of the commercially available flow diverter Derivo® Embolization Device (DED, Acandis GmbH, Germany), which is intended for the treatment of cerebral aneurysms of vessel diameters from 2.5 mm up to 6 mm, geometrical characteristics of which are presented in previous preclinical and clinical literature [9]. Samples were manufactured and provided by Admedes GmbH (Germany). Consisting of 24 Nitinol wires (Fort Wayne Metals, USA) with a thickness of 40 µm and a braiding angle (between wire and implant axis) of 75°, Nitinol wires were bent and braided backwards at the distal implant end, resulting in 48 wires at implant cross section and open wire ends proximally. Samples were 4.0 × 25 mm, with a 25° flaring region at both implant ends.

In the electropolished samples, wires were electropolished before the braiding process, leading to material removal according to internal process specifications. These samples correspond to the commercially available and clinically used DED, presenting a treated surface with blue colour defined as BlueXide®. As control, samples with the same geometry but not electropolished native Nitinol wires were manufactured. Wire diameter was equal to the final wire diameter after electropolishing in the first group. After braiding and cutting of the wires, all samples underwent annealing following standard processes for Nitinol form setting, in a salt bath at 500 ± 50 °C for less than 10 min.

For protein adsorption, endothelialisation and immunogenicity testing Nitinol-discs with a diameter 10 mm and a thickness of 0.2 mm were used because a flat substrate was required. Samples were subjected to the same process described above, starting from native Nitinol disk, electropolishing a part of the discs and submitting all electropolished and native disks to annealing with the same conditions as described above.

### Chemical surface characterization

Auger electron spectroscopy (AES) was performed for surface characterization using Nitinol sheets. One specimen with electropolished/blue oxide surface subjected to the same process described above and one specimen with native surface were analysed at Federal Institute for Materials Research and Testing, Berlin, Germany, using an ULVAC-PHI Scanning Auger Nanoprobe PHI 700.

AES depth profile was referenced with a scan rate of 59.3 nm/min using a BCR-261T Ta-Sheet. Depth of the titanium oxide layer was defined as the point at which titanium signal strength in Nitinol exceeds oxygen signal strength.

In two further specimens, scanning electron microscopy (SEM) was performed for qualitative assessment of surface quality (JEOL JSM-6610 Scanning Electron Microscope, JEOL Ltd, Japan).

### Corrosion behaviour testing

Corrosion testing of braided neurovascular devices was performed according to ASTM F2129-08 by conducting potentiodynamic polarization using a working electrode (braided sample as described above), a reference electrode (e.g. saturated calomel electrode (SCE)) and an auxiliary electrode (a noble material, usually platinum or graphite) in phosphate-buffered saline (PBS) at body temperature. Computer-controlled electrochemical test systems consisting of Voltalab PGP201 (Radiometer Copenhagen Analytical S.A., France) and Iviumstat (Ivium Technologies, The Netherlands) potentiostats equipped with Voltamaster and IviumSoft software were used to perform the corrosion measurements. Typical results of the outlined test set up are the rest potential, which is the potential of the unpolarized working electrode relative to the reference electrode, and the breakdown potential, which is the potential where pitting or crevice corrosion or both will initiate and propagate [10].

### Focused ion beam (FIB)

Focused ion beam (FIB) imaging was performed on braided samples with electropolished, blue oxide surface to visualize oxide layer thickness and application using a Zeiss Crossbeam 540 (Carl Zeiss AG, Germany) in collaboration with Aalen University (Aalen, Germany). FIB was used to gently remove the targeted area and to create a material cross section, which could be imaged by SEM subsequently.

### Nickel release

The amount of Ni release per device was investigated in accordance with ISO 10993-18 in collaboration with MDT Medical Device Testing GmbH, Germany. Specimens were fractionally extracted at 37 ± 2 °C for 1, 2, 3, 4, 5, 6, 7, 10, 15, 20, 25, 30, 35, 40, 45, 50, 55 and 60 days. After the extraction, each specimen was investigated with a Thermo, ICAP 6300, using an inductively coupled plasma (ICP) spectrometer to determine the amount of Ni release [11].

### Blood sampling

Blood sampling procedures were approved by the ethics committee of the University of Tuebingen, Germany. Blood was collected by venipuncture from volunteers, who provided signed informed consent. To guarantee optimal haemostatic function, blood donors, who took haemostasis-affecting agents, like acetylsalicylic acid; oral contraceptives; nonsteroidal antiphlogistics; and others, were excluded. Blood was anticoagulated with Heparin-Natrium (Rathiopharm GmbH, Germany). All subjects were free of platelet-affecting drugs for at least 14 days to ensure optimal haemostatic function.

### Protein adsorption

Electropolished/blue oxide or native Nitinol test specimen were incubated with 2 ml heparinized fresh human whole blood (*n* = 3; 1.5 IU/ml heparin) for 1 h at 37 °C on a rocking platform. Differences in the adhesion of two main blood plasma proteins were detected using a specially designed enzyme-linked immunosorbent assays (ELISA) method as previously described [12]. Briefly, the samples were fixed for 1 h with 4% paraformaldehyde (PFA) buffer at 4 °C and blocked overnight. Surface-bound fibrinogen or albumin was detected using a specific primary goat anti-human fibrinogen (Sigma Aldrich, Germany) or a sheep anti-human albumin (BioRad, Germany) antibody, respectively, followed by incubation with an alkaline phosphatase-conjugated secondary antibody (Immunotech/Coulter, France) and a chromogenic pNPP substrate (Sigma Aldrich, Germany). The reaction was stopped by addition of NaOH, and light absorbance was measured at 405 nm with the BioTek EON™ photometer (BioTek EON™, USA).

### In vitro thrombogenicity model

In order to compare the hemocompatibility of electropolished/blue oxide and native Nitinol stents, a well-established thrombogenicity model was used to simulate blood circulation at 37 °C [13].

For each blood donor (*n* = 5), baseline values were measured directly after blood sampling in 6 ml blood, which was heparinized with 1.5 IU/ml during blood withdrawl. Three polyvinyl chloride tubes with 75-cm length and 3.2-mm inner diameter (Saint-Gobain Performance Plastics, France), previously coated with heparin by Ension (Ension Inc, USA), were either left empty or loaded with either one electropolished or one native stent. Subsequently, each tube was filled with a total of 6 ml heparinized human blood. Afterwards, each tube was closed into a circuit by silicone connection tubing, and the blood was circulated in a water bath at 37 °C and 150 ml/min for 60 min using a peristaltic pump (Cole-Parmer GmbH, Germany).

### Whole blood count analysis

After blood sampling (baseline) and after circulation, each blood sample was anticoagulated using EDTA (EDTA-Monovette, Sarstedt, Nuembrecht, Germany) for blood count analysis using an ABX Micros 60 blood analyser (Axon Lab AG, Switzerland).

### Measurement of haemostatic markers

Before and after circulation, blood was sampled into tubes containing corresponding terminating media and centrifugated, followed by blood plasma collection and storage at −20 °C or −70 °C until the analysis of hemocompatibility markers using ELISA. Analyses of thrombin–antithrombin (TAT) complex and Sc5b-9 concentrations were performed using ELISA kits from Siemens Healthcare (Germany) and TECOmedical (Germany), respectively.

### Scanning electron microscopy (SEM)

SEM was performed to investigate the rate of platelet adhesion and fibrin network formation on the surface of the electropolished and native stents after circulation in the thrombogenicity model, as described previously [13].

### Endothelialization model

Prior to cell cultivation, electropolished/blue oxide or native Nitinol test specimen were preincubated for 10 min at 37 °C under soft agitation with 1-ml platelet-rich plasma (PRP). PRP was prepared from fresh human whole blood, which was anticoagulated with 3 IU/ml Heparin, by centrifugation for 10 min at 160 *g* and room temperature. After washing with PBS, each plate was put into a well of a 12-well plate and seeded with 120,000 human umbilical vein endothelial cells (HUVECs) in 1 ml VascuLife EnGS medium (Lifeline Cell Technology, USA) containing VascuLife EnGS LifeFactors Kit, 50 µg/ml gentamicin and 0.05 µg/ml amphotericin B (PAA Laboratories, Germany). The samples were incubated for 48 h at 37 °C and 5% CO_2_. The HUVECs were isolated as previously described [14].

After incubation, the samples were washed with PBS, fixed using CellFix (BD Biosciences, Germany) and subsequently permeabilized in 90% ice-cold methanol. After washing, DAPI nuclear dye (300 nmol) was incubated for 3 min and the samples were investigated using a fluorescence microscope (Optiphot-2, Nikon, Germany) equipped with a DSLR remote control (Nikon 550 D).

### Real-time qPCR

The immune reaction of the cells that might be potentially triggered after contact with electropolished/blue oxide or native Nitinol was investigated using real-time qPCR. Again, plates were preincubated with PRP and seeded with HUVECs as described above. After an incubation time of 48 h, total RNA was isolated from the cells using the Aurum total RNA isolation kit (Bio-Rad). The isolated RNA was converted to copyDNA (iScript kit, Bio-Rad). Expressions of Intercellular Adhesion Molecule 1 (ICAM-1), E-selectin, interleukin-1 (IL-1) and IL-6 were analyzed using the CFX Connect teal-time PCR detection system (Bio-Rad). GAPDH expression was used as reference, and TNF-α activated cells (5 ng/ml) were used as positive controls to guarantee cell functionality.

### Statistics

All analyses were performed using the statistical software package GraphPad Prism (version 5, GraphPad Software, USA). The Kolmogorov–Smirnov test was used to test for normal distribution. Normally distributed data are shown as mean ± SD and not-normally distributed data are shown as median with range. Differences between normally distributed data of two groups were assessed using paired Student’s *t* tests. Normally distributed data consisting of three or more groups are shown as mean ± SD and were analyzed using repeated-measures ANOVA with Tukey’s multiple comparisons test to analyze differences between groups. Not-normally distributed data were analyzed using Friedman test with Dunn’s multiple comparisons test. Statistical significance was defined as *p* < 0.05.

## Results

### Surface characterization

**Fig. 1:**
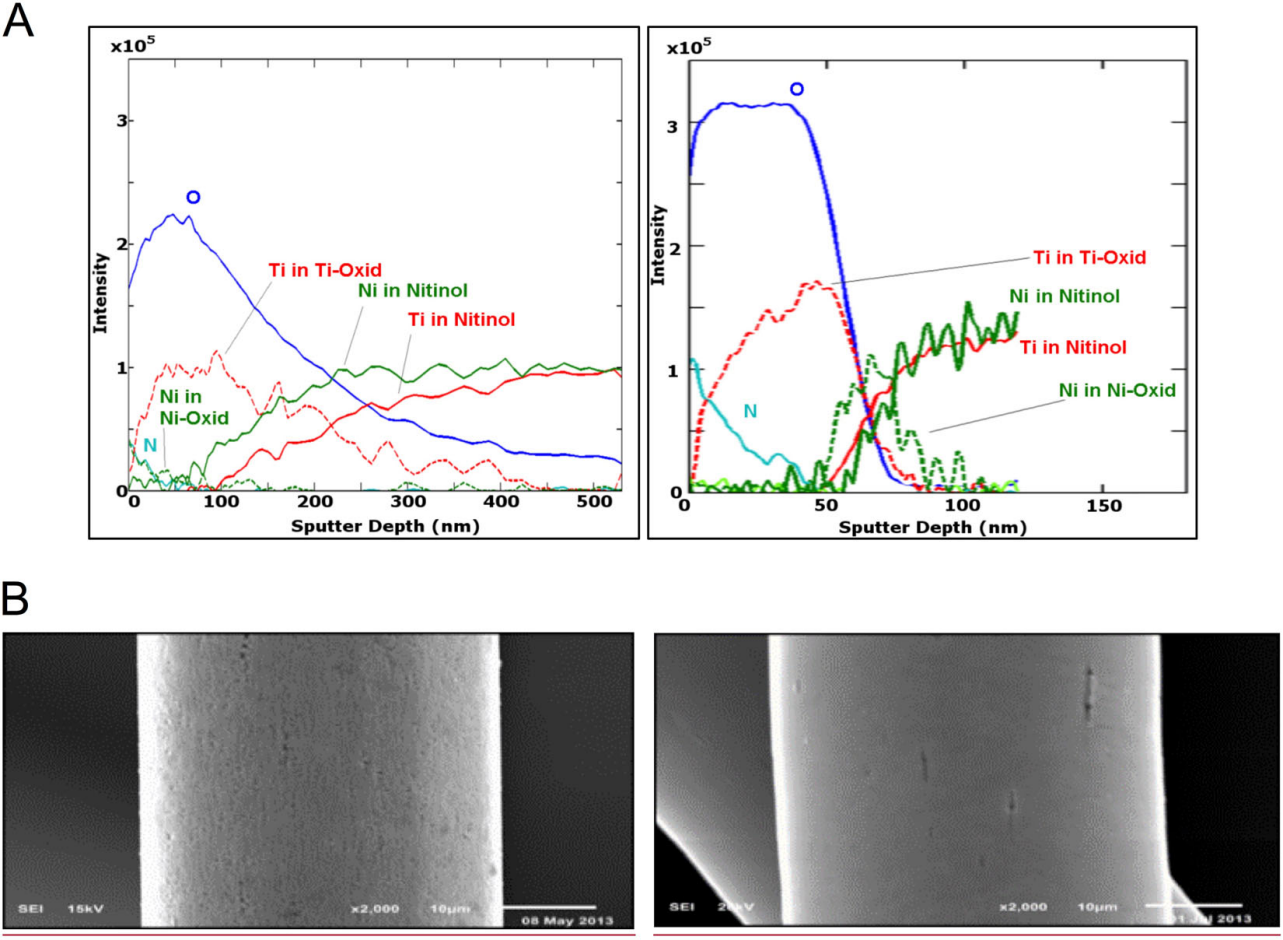
**a** Chemical composition detected with Auger electron spectroscopy for samples with native oxide (left) and electropolished/blue oxide surface (right). **b** Scanning electron microscopy images of a Nitinol wire with native oxide surface (left) and electropolished/blue oxide surface (right)

Chemical composition detected using AES was considerably different in both samples with electropolished/blue oxide and native oxide surface (Fig. 1a). On electropolished Nitinol sheets with the blue oxide surface (right graph) a high oxygen intensity with a pronounced plateau in the superficial 50 nm layer was observed. Oxygen intensity rapidly decreased at around 60 nm depth to the level of titanium intensity, corresponding to oxide layer thickness of about 60 nm according to the definition above. In addition to oxygen, a strong nitrogen signal was present in the first 50 nm from the surface. On the native oxide surface (left graph), oxygen intensity was significantly lower and thickness of the layer corresponding to the intersection with the titanium intensity was about 250 nm. Nitrogen intensity was much lower in the native oxide compared to the blue oxide. As a further difference, we observed relevant nickel signals in the native oxide, while nickel intensity in the blue oxide surface was close to the intensity detection limit. Nickel intensity in the oxide layer was considerably reduced in electropolished Nitinol sheets compared to that in native oxide.

SEM imaging of the different wires showed a smoother surface on the electropolished/blue oxide wires (Fig. 1b right image) with some remaining single localized grooves resulting from dissolution of non-metallic inclusions, whereas native oxide surface (Fig. 1b left image) presented an overall rough and grooved topography resulting from wire manufacturing and annealing processes.

### Corrosion

**Fig. 2:**
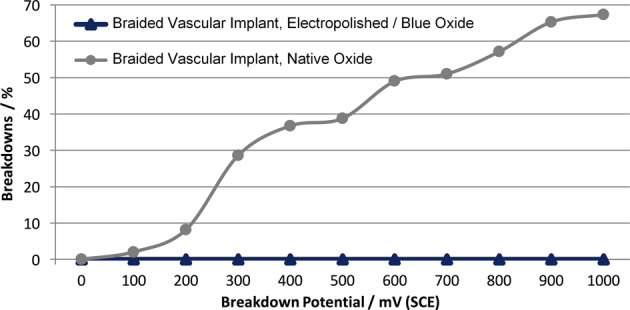
Percentage of breakdown versus threshold potential for two groups of braided neurovascular devices. Electropolished/blue oxide surface (*n* = 117) and native oxide surface (*n* = 49) samples

In Fig. 2 the percentage of breakdown versus threshold potential for the two different groups of braided neurovascular devices (with blue oxide and native oxide surface, respectively) are shown. As a result of the potentiodynamic polarization measurement performed per ASTM F2129-08, there were zero (0) breakdowns detected for the blue oxide group, whereas native oxide devices exhibited a high number of breakdowns even at low potentials.

### Nickel release

**Fig. 3:**
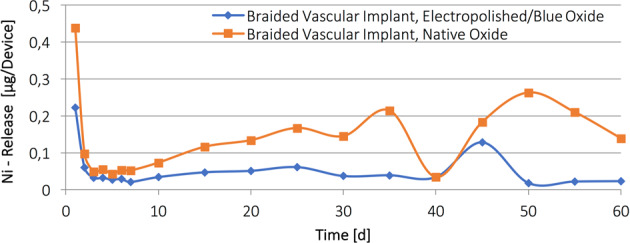
Nickel release data in µg/device over days for braided vascular implants with electropolished and native oxide surfaces

Figure 3 shows the results of the performed nickel release testing for braided vascular implants with blue oxide and native oxide surface. Specimens with blue oxide surface do show less Nickel release at first day, for short term spread (days 2–7) and throughout the remaining evaluation (days 10–60) compared to the specimens with native oxide surface.

### FIB

**Fig. 4:**
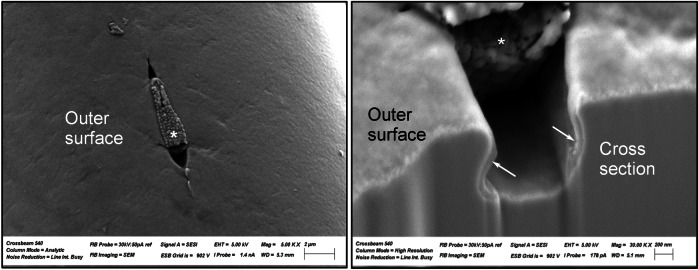
Scanning electron microscopy image of a particle void assembly taken from a single 42-µm-thick Nitinol wire of a braided neurovascular device with electropolished/blue oxide surface finish (left) and the corresponding cross-sectioned interface applied via focused ion beam (right). Asterisk shows the particle void assembly on the material surface. Arrows show the oxide layer covering the surface surrounding the inclusion

Figure 4 visualizes the applied blue oxide with its characteristic thickness of around 60–70 nm. This oxide covers not only the exposed outer surface, but also the void next to a particle inclusion at the surface. It can be concluded from these results that the applied blue oxide layer shows the ability to recover surface inhomogeneities such as PVAs inclusions and, therefore, contributes to superior behaviour regarding Ni-Release and corrosion resistance, as discussed later.

### Adsorption of plasmatic albumin and fibrinogen

**Fig. 5:**
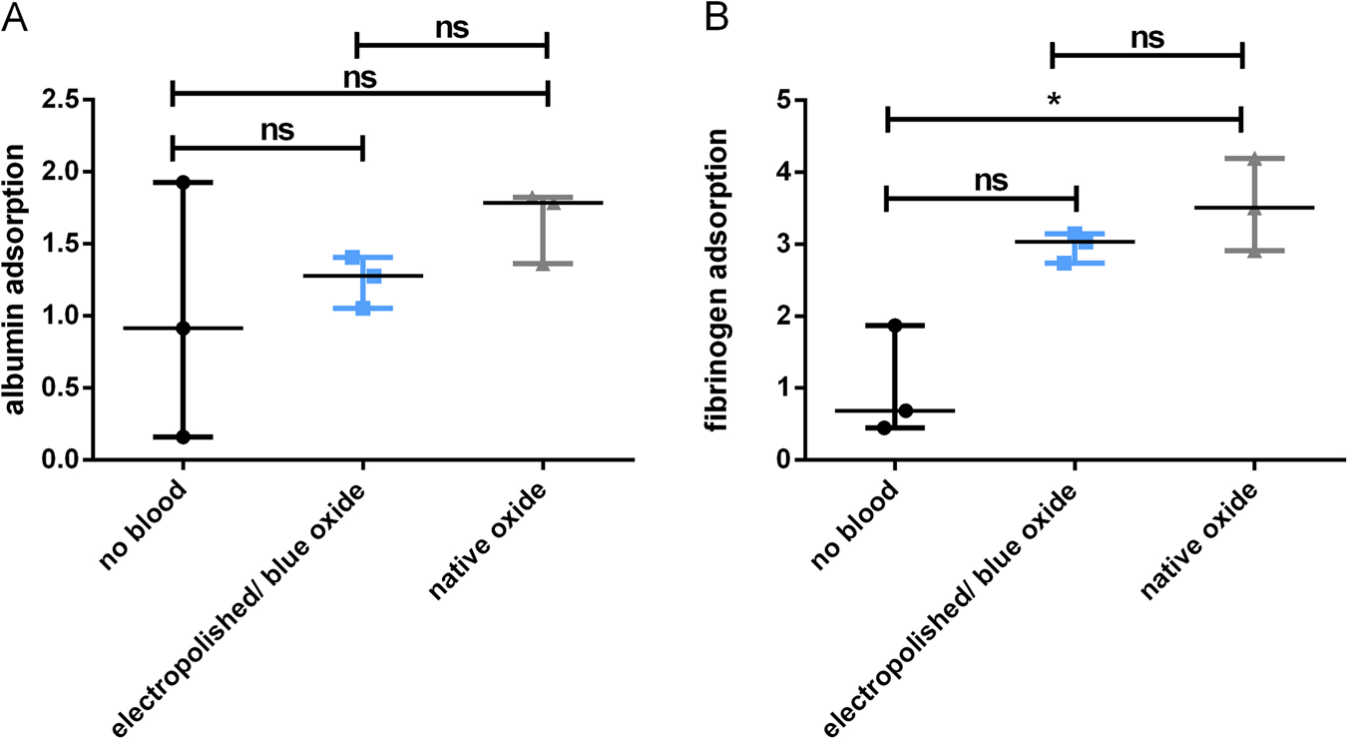
Detection of albumin (**a**) and fibrinogen (**b**) adsorption on electropolished and native Nitinol test specimens after incubation with fresh human blood for 1 h at 37 °C under soft agitation. Test specimen without blood contact served as reference, and data measured in the native and electropolished surface groups are given in relation. Data are shown as medians and range (*n* = 3). ns not significant, *p < 0.05

In a first experimental set-up, potential differences in adsorption of two main plasma proteins on the two different surfaces were analyzed. The mean values of albumin and fibrinogen adsorption on the electropolished/blue oxide and native surface are shown in Fig 5. Compared to the electropolished surface, the amounts of adsorbed albumin (Fig. 5a) and fibrinogen (Fig. 5b) on the native surface are higher, indicating a trend towards the reduction of protein adsorption due to the electropolished surface.

### Hemocompatibilty testing of electropolished and native Nitinol surfaces

**Fig. 6:**
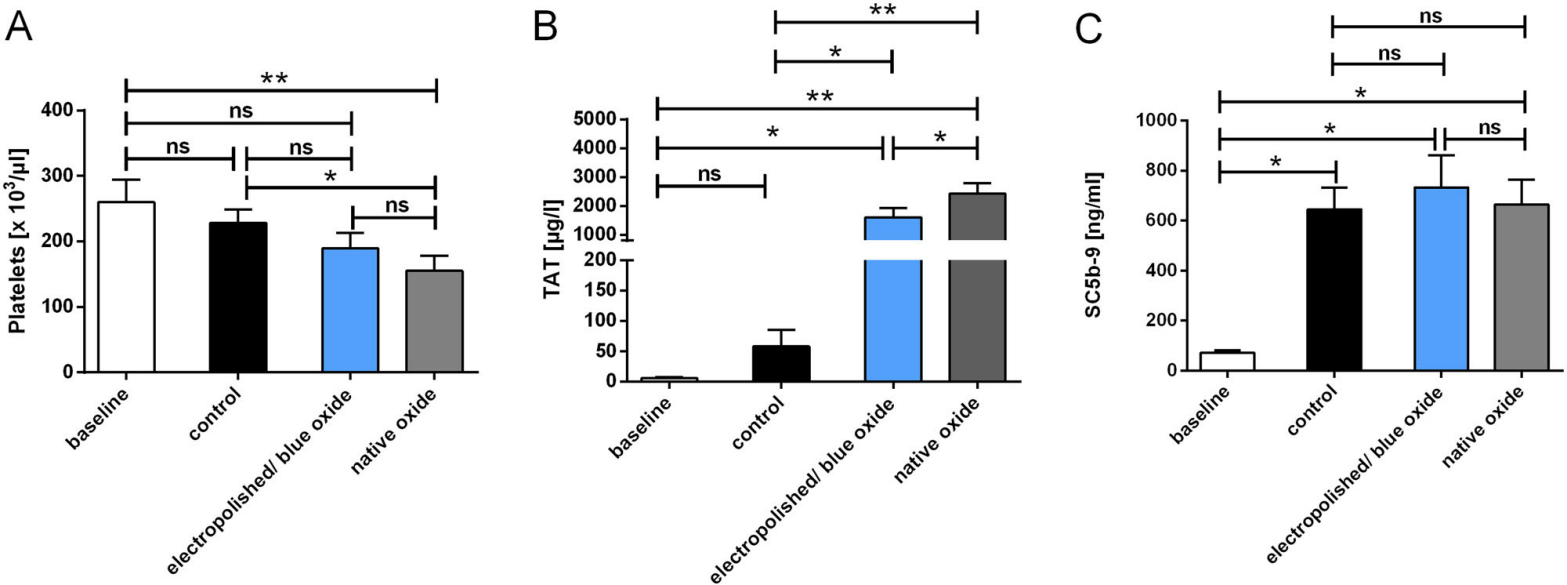
Platelet counts (**a**), blood coagulation (**b**; thrombin–antithrombin (TAT)) and the complement system (**c**; SC5b-9) were quantified in untreated human whole blood (baseline), blood circulated in empty tubing (control) or in blood circulated in tubing containing electropolished/blue oxide or native Nitinol stents using ELISA. The Kolmogorov–Smirnov test was used to test for normal distribution. Data are shown as mean ± SD (*n* = 5) and were analyzed by repeated-measures ANOVA with Tukey’s multiple comparisons test. ns: not significant, *p < 0.05, **p < 0.01

After implantation, the stent gets into contact with the patient’s blood, which can lead to different biochemical reactions and may have an effect on the haemostatic system. To analyze the hemocompatibility of the two different surfaces, a well-established thrombogenicity model was used [13]. During circulation, electropolished stents did not alter the number of platelets (Fig. 6a), white blood cells (Supplementary Fig. 1a) and red blood cells (Supplementary Fig. 1b) as well as haemoglobin concentrations (Supplementary Fig. 1c) and haematocrit levels (Supplementary Fig. 1d) when compared to the control group. In contrast, a significant decrease in platelet counts was detected in the native surface group, when compared to baseline values (before circulation) and the control group (circulation with blood only).

Moreover, activating effects on the coagulation cascade and the complement system were determined by measuring the concentration of thrombin–antithrombin III (Fig. 6b) and SC5b-9 (Fig. 6c) complexes. The baseline values were again those measured in untreated human blood.

Compared to a TAT concentration of 57.7 µg/l in the control group, the electropolished stents (1609.3 µg/l, *p* < 0.05) and the native stents (2434 mg/l, *p* < 0.01) showed significantly increased TAT levels. However, the activation of the coagulation cascade was more pronounced in the native stent group when compared to the electropolished stent group (*p* < 0.05).

SC5b-9, the final complex of the complement system, significantly increased after circulation in the control group (645 ng/ml, *p* < 0.05), the electropolished stent group (733 ng/ml, *p* < 0.05) and the native Nitinol stent group (664 ng/ml, *p* < 0.05) compared to baseline levels of 72 ng/ml.

Platelet adhesion as well as the formation of fibrin networks on the electropolished and native Nitinol stent surfaces was examined using SEM. The results show that the adhesion of platelets and fibrin network formation is donor-specific (Supplementary Fig. 2). Due to the donor-specific number of platelets and other blood proteins, the images of the five different donors cannot be compared with each other. There are also no clearly visible differences between both surfaces, although the quantitative results of the whole blood count analysis clearly showed that the number of platelets decreased significantly after contact with the native stent surface (Fig. 6a).

### Endothelialisation properties

**Fig. 7:**
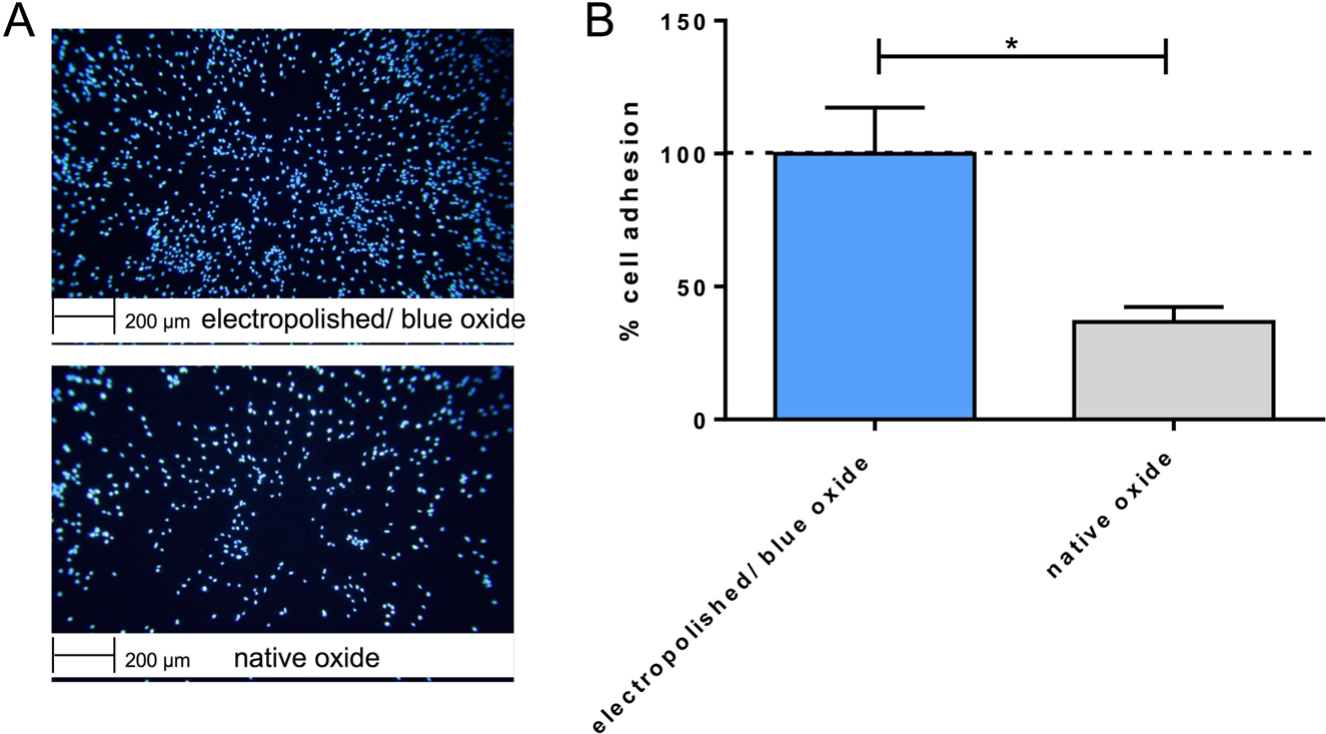
**a** Representative images of DAPI-stained human umbilical vein endothelial cells (HUVECs) cultivated on electropolished/blue oxide and native Nitinol scaffolds, which were preincubated with fresh human platelet-rich plasma, for 48 h at 37 °C and 5% CO_2_. **b** Statistical analysis of cell numbers counted 48 h after seeding HUVECs on the on electropolished/blue oxide and native Nitinol scaffolds (*n* = 4). Mean values of the electropolished surface groups were transformed to 100% and data measured in the native surface groups are given in relation. Data are shown as mean ± SD (*n* = 4) and were analysed by one-tailed paired *t*-test. *p < 0.05

The endothelialisation properties were analysed using Nitinol discs due to easier handling and with the aim to determine potential differences of the surfaces without the impact of the stent design. The discs were preincubated with fresh human PRP obtained from four different donors to simulate the first physiological contact of plasma proteins and platelets after implantation of a stent. Subsequently, HUVECs were cultivated for 48 h on the surface to analyse the endothelialisation properties. After cultivation, the number of cells on each surface was determined for a quantitative comparison (Fig. 7a). The mean values of the electropolished/blue oxide group were transformed to 100%; there is a significant relative decrease in cell adhesion to 36.7% (*p < 0.05) on the native Nitinol surface (Fig. 7b).

### Immunogenicity testing

**Fig. 8:**
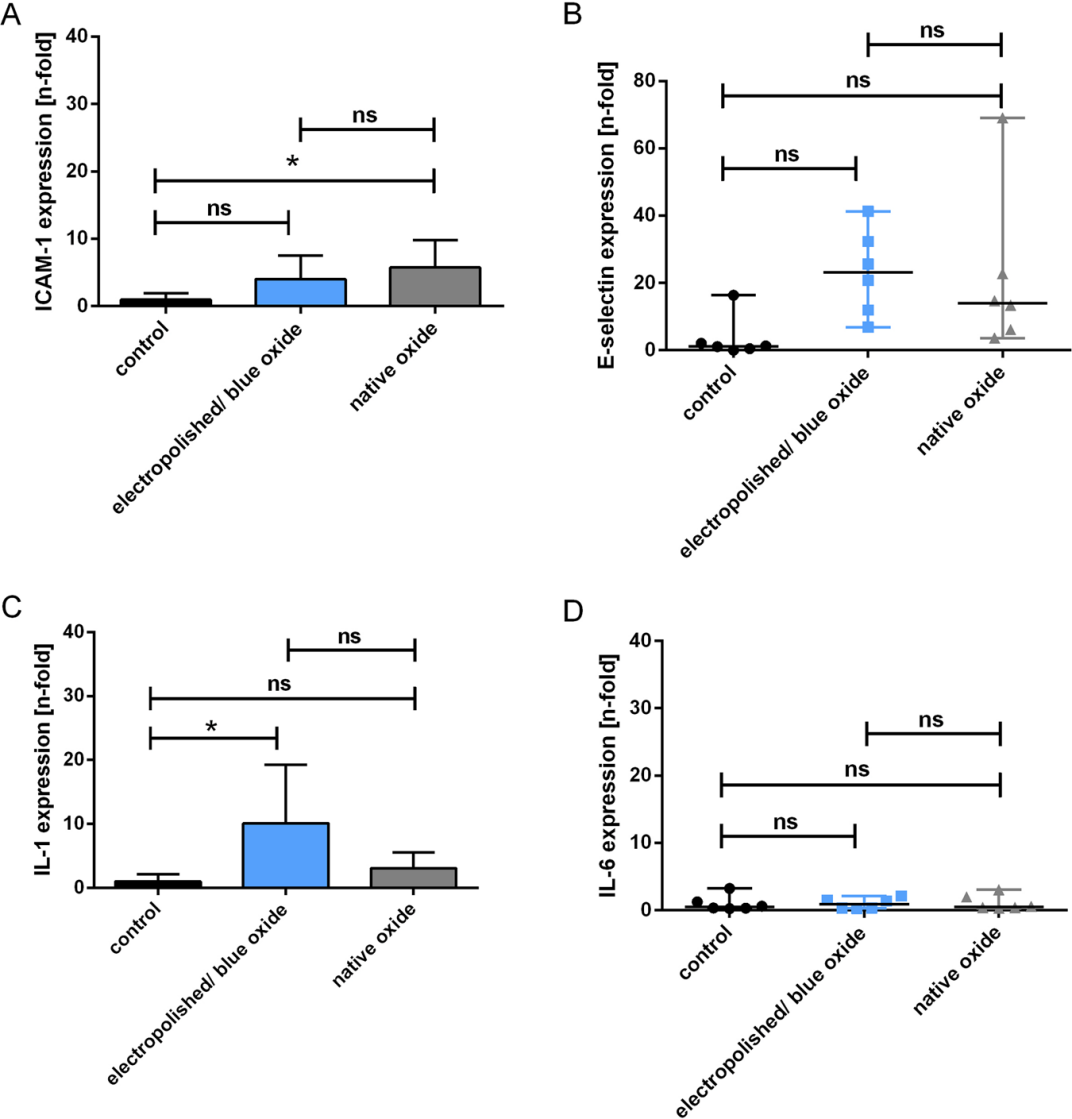
Investigations of immunogenic effects of the electropolished/blue oxide and native surface on primary ECs. Relative normalized gene expressions of ICAM-1 (**a**), E-selectin (**b**), IL-1 (**c**) and IL-6 (**d**) in HUVECs after 48 h incubation on electropolished and native Nitinol plates was measured using real-time qPCR. Unstimulated and TNF-α-activated HUVECs cultivated in tissue culture plates served as negative and positive controls, respectively, to confirm cell functionality. The Kolmogorov–Smirnov test was used to test for normal distribution. Normally distributed data are shown as mean ± SD and were analyzed using repeated-measures ANOVA with Tukey’s multiple comparisons test to analyze differences between groups (**a**, **c**). Not-normally distributed data are shown as median with range and were analyzed using Friedman test with Dunn’s multiple comparisons test (**b**, **d**). (*n* = 6). ns not significant, *p < 0.05

Results of real-time qPCR analysis are shown in Fig. 8. The expression of ICAM-1 (Fig. 8a) significantly increases, almost 6-fold, after contact with the native Nitinol surface. In the TNF-α-stimulated control group, the ICAM-1 expression was increased 323-fold (data not shown) compared to the unstimulated control group.

E-selectin expression (Fig. 8b) increased after contact with both surfaces, showing no significant difference between the both surfaces. Again, in the TNF-α-stimulated control group the E-selectin expression was significantly increased, around 2891-fold (data not shown).

IL-1 expression in HUVECs significantly increases, 10-fold, after incubation with the electropolished surface (Fig. 8c) and 1682-fold in the positive control group (data not shown). There was no change in the IL-6 expression after the contact with both surfaces, whereas TNF-α-stimulation increased IL-6 expression by around 4.3-fold (data not shown).

## Discussion

Braiding technology is a promising tool for the manufacturing of endovascular Nitinol implants in a broad field of biomedical indications, particularly for applications where high adaptation to vessel anatomy and fine-meshed structures are required. Whereas these properties make braided implants attractive in the field of neurovascular intervention, the small-vessel lumens, between 2 and 5 mm in diameter, in intracranial arteries commonly affected by intracranial aneurysms together with the devastating effect of a potential flow impairment place the highest demands on surface biocompatibility. Neurovascular flow diverters, with a high metal density aimed at blood flow reduction within the aneurysm sack, are related to ischaemic complications, including distal embolism, in-stent thrombosis and stenosis in the clinical literature [15, 16]. Besides implant design and wrong vessel apposition, which can potentially affect the thrombogenicity of endovascular implants [17, 18], material and surface characteristics are well-known determinants of blood and vessel wall reaction and, thus, of the implant biocompatibility [19, 20].

Electropolishing is a well-established technology for producing biocompatible surfaces of super-elastic Nitinol laser-cut implants. In Sullivan et al. [21], stent electropolishing resulted in a 4 nm oxide layer, which was correlated to a lower nickel release and higher corrosion resistance compared to unpolished stents.

In our work, we present an in vitro investigation of a fine-meshed, braided Nitinol implant for neurovascular flow diversion manufactured by means of a novel electropolishing/blue oxide technology: in contrast to laser-cut stents, where electropolishing represents the last manufacturing step, wires were electropolished before braiding and final annealing for shape setting. We characterized the surface and performed in vitro biological evaluation, comparing specimens with the native oxide and electropolished/blue oxide surface.

As expected, AES revealed a considerably thinner oxide layer in the electropolished/blue oxide samples compared to the native oxide Nitinol. The electropolished/blue oxide surface exhibited considerably higher oxygen and nitrogen and lower nickel signals in the oxide layer compared to the native oxide surface. We assume the thin titanium oxynitride or titanium oxide + titanium nitride layer to be protective against corrosion and nickel release, since both were considerably reduced in blue oxide specimens compared to native oxide samples. These results are in basic accordance with the observation of Clarke et al. [22], who showed that, in electropolished Nitinol, a thinner oxide layer was correlated to lower nickel concentration and even to lower nickel release and corrosion.

We concentrated on the effect of both native oxide and electropolished/blue oxide surfaces in contact with biological tissue. We investigated the hemocompatibility in blood flow directly on the braided implants and produced disks via the same manufacturing steps for evaluation of protein adhesion, cell adhesion and proliferation and inflammatory marker expression.

Since the formation of protein layers on the endovascular implant surface has the potential to influence subsequent biochemical processes including haemostasis, inflammation and cell proliferation, we measured the adsorption of albumin, the most abundant protein in blood, as well as fibrinogen, a central protein in blood clotting [23]. Though we did not show a significant difference between both surfaces, a trend toward lower protein adsorption in electropolished/blue oxide samples was observed.

For in vitro evaluation of the thrombogenicity, the blood flow rate and vessel geometry used in the flow loop model were selected based on the physiological condition of a large intracranial vessel, such as the media cerebral artery [24]. Using TAT as a sensitive coagulation marker, we observed a significantly lower value in electropolished/blue oxide compared to native Nitinol braided samples. In the same tests, a trend for lower platelet adhesion on electropolished/blue oxide wires was observed, with only blood platelet count in the native wire group being significant lower than in the control line without implant. Since the geometry of the implants in both groups was identical, the decrease of coagulation activity in the electropolished/blue oxide specimens can be attributed to the different surface characteristics. We suppose that lower nickel ion release and smoother surface could play a role in reducing protein and platelet adhesion as well as decreasing activation of the coagulation cascade. In a previous study, high nickel concentrations but not surface roughness correlated with higher albumin adsorption on nitinol wires [25]. Moreover, in an in vitro investigation, higher roughness of thin-film nitinol samples led to an increase not only of protein adsorption but also of cell adhesion and thrombotic response [26]. However, SEM analysis showed no clearly visible differences in fibrin and platelet adhesion between both groups, which is probably mainly due to the high fluctuation among the five donors.

For endothelial cell proliferation and inflammatory marker expression, specimens were preincubated in fresh human PRP before cell contact, simulating the blood contact before vessel interaction for the reason described above. Cell proliferation on disks with electropolished/blue oxide surface was significantly higher than on native oxide surface. We cannot state whether the different protein adsorption profiles or the material itself were responsible for these results, since we did not perform the investigation with a control group without previous RPR contact. However, previous in vitro studies suggest possible factors, which are directly related to surface chemistry and physics. First, endothelial cell adhesion was enhanced by high nitrogen concentration at the surface of stainless steel 316 L in stents for coronary artery treatment [27]. Also in the electropolished/blue oxide specimens tested in this study, nitrogen concentration formed during annealing after electropolishing was considerably higher than in native oxide. Second, nickel release could be detrimental for cell proliferation. Plant et al. [28] showed an increased oxidative stress within HUVECs in contact with a not-polished, nickel-rich Nitinol surface, whereas this pathological process was eliminated by displacing nickel ions from the metal surface. Moreover, a cytotoxic effect of nickel concentration was also proven in fibroblast culture [29] and epithelial cell culture [30]. As a last possible surface factor, lower surface roughness (which was qualitatively assessed in our study by means of SEM) was previously proven to increase endothelial cell adhesion and proliferation [31].

Investigation of four essential inflammatory markers possibly induced by endothelial cells upon contact with electropolished/blue oxide or native Nitinol did not find significant differences between surfaces. However, there was a trend to lower ICAM-1 levels and a significant increase in IL-1 expression for electropolished surfaces. These results need to be confirmed and clearly elucidated by further studies focusing on the investigation of potential proinflammatory effects on endothelial cells induced by both surfaces.

A last observation concerns the delivery force of braided implants, which is reduced by electropolishing in our experience (internal data, not published). A possible reason can be found in the higher nitrogen content demonstrated on AES. In this context, we refer to Liu et al. [32], who demonstrated a reduction of friction coefficient for nitrogen-plasma-implanted Nitinol surfaces.

In conclusion, we presented in vitro investigation of a new braided implant generation, where direct electropolishing of single Nitinol wires was performed before braiding and final annealing. We extensively characterized the resulting blue oxide surface and showed a significant improvement in terms of thrombogenicity and endothelial cell proliferation compared with not-electropolished Nitinol with native oxide surface.

Finally, both properties have the potential to improve outcomes of aneurysm treatment in clinical settings. A low-thrombogenic behaviour potentially allows for a reduction of thrombo-ischaemic complications like in-stent-thrombosis, side branch occlusion or distal embolism, improving the safety of the treatment. On the other hand, an improved endothelialisation is fundamental for an effective and lasting reconstruction of the vessel and, thus, exclusion of the aneurysm from the circulation, the final target of the therapy.

## Supplementary information

Supplementary FigureS1

Supplementary FigureS2

Supplementary legends

Electronic Supplementary material
