# Publisher Correction: In vitro investigation of chemical properties and biocompatibility of neurovascular braided implants

**DOI:** 10.1007/s10856-020-06447-w

**Published:** 2020-11-07

**Authors:** Giorgio Cattaneo, Chris Bräuner, Gerd Siekmeyer, Andreas Ding, Sabina Bauer, Markus Wohlschlögel, Lisa Lang, Teresa Hierlemann, Maria Akimov, Christian Schlensak, Andreas Schüßler, Hans-Peter Wendel, Stefanie Krajewski

**Affiliations:** 1 Acandis GmbH, Pforzheim, Germany; 2 Admedes GmbH, Pforzheim, Germany; 3 Acquandas GmbH, Kiel, Germany; 4 Department of Thoracic and Cardiovascular Surgery, Clinical Research Laboratory, University Medical Center, Tuebingen, Germany

**Correction to:** Journal of Materials Science: Materials in Medicine (2019) 30:67

10.1007/s10856-019-6270-6

The article “In vitro investigation of chemical properties and biocompatibility of neurovascular braided implants”, written by Giorgio Cattaneo, Chris Bräuner, Gerd Siekmeyer, Andreas Ding, Sabina Bauer, Markus Wohlschlögel, Lisa Lang, Teresa Hierlemann, Maria Akimov, Christian Schlensak, Andreas Schüßler, Hans-Peter Wendel and Stefanie Krajewski, was originally published Online First without Open Access. After publication in volume 30, issue 6, article number 67 the author decided to opt for Open Choice and to make the article an Open Access publication. Therefore, the copyright of the article has been changed to © Ownership of copyright in the article shall vest in the author 2020 and the article is forthwith distributed under the terms of the Creative Commons Attribution 4.0 International License, which permits use, sharing, adaptation, distribution and reproduction in any medium or format, as long as you give appropriate credit to the original author(s) and the source, provide a link to the Creative Commons licence, and indicate if changes were made. The images or other third party material in this article are included in the article’s Creative Commons licence, unless indicated otherwise in a credit line to the material. If material is not included in the article’s Creative Commons licence and your intended use is not permitted by statutory regulation or exceeds the permitted use, you will need to obtain permission directly from the copyright holder. To view a copy of this licence, visit http://creativecommons.org/licenses/by/4.0.

The original article has been corrected.

