# Supplementary figures and images for: In vitro investigation of chemical properties and biocompatibility of neurovascular braided implants
Source: J Mater Sci Mater Med. 2019 Jun 4;30(6):67. doi: 10.1007/s10856-019-6270-6 (PMC7695648; doi:10.1007/s10856-019-6270-6)

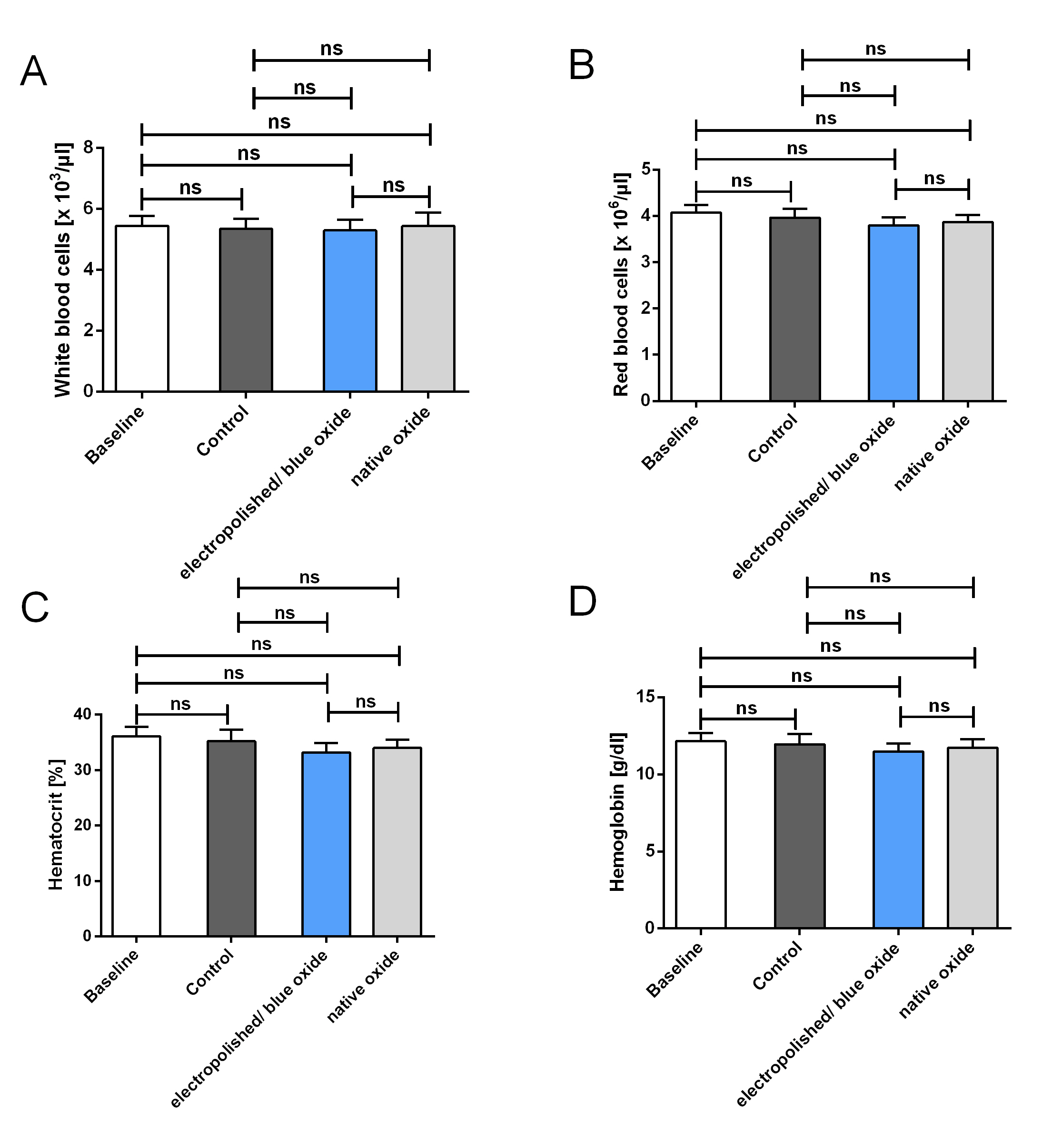

Supplement: Supplementary file 1 — Supplementary FigureS1 [file 10856_2019_6270_MOESM1_ESM.jpg]

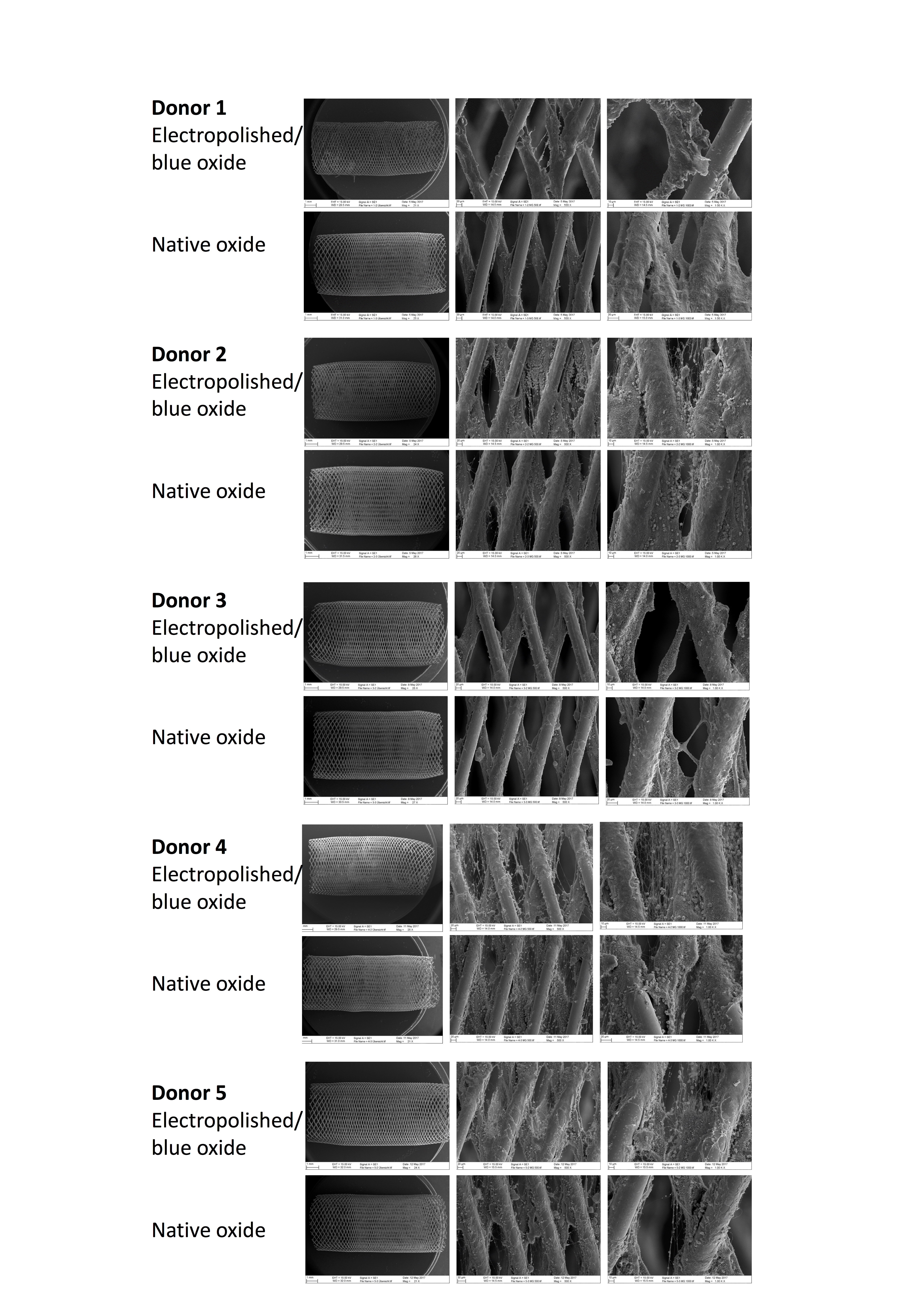

Supplement: Supplementary file 2 — Supplementary FigureS2 [file 10856_2019_6270_MOESM2_ESM.jpg]
