# Supplementary material for: In vitro investigation of chemical properties and biocompatibility of neurovascular braided implants
Source: J Mater Sci Mater Med. 2019 Jun 4;30(6):67. doi: 10.1007/s10856-019-6270-6 (PMC7695648; doi:10.1007/s10856-019-6270-6)
Supplement: Supplementary file 3 — Supplementary legends [file 10856_2019_6270_MOESM3_ESM.docx]

**Supplementary legends**:

**Supp Figure 1:** Electropolished stents do not change white blood cell counts (a), red blood cell counts (b), hemoglobin concentration (c) and hematocrit levels (d) after 60 min of circulation at 150 ml/min with fresh human whole blood. Data are shown as mean ± SEM (n = 5) and were analyzed by repeated-measures ANOVA with Tukey`s multiple-comparison test. ns: not significant

**Supp Figure 2:** Scanning electron microscope images of electropolished and native Nitinol stents after 60 min of circulation at 150 ml/min with fresh human whole blood. Pictures form all five donors are shown.
